# Supplementary material for: Cingulate cortex shapes early postnatal development of social vocalizations
Source: bioRxiv. 2024 Dec 2:2024.02.17.580738. Originally published 2024 Feb 21. Preprint. [Version 2] doi: 10.1101/2024.02.17.580738 (PMC10962701; doi:10.1101/2024.02.17.580738)
Supplement: Supplement 1 [file NIHPP2024.02.17.580738v2-supplement-1.pdf]

bioRxiv preprint doi: https://doi.org/10.1101/2024.02.17.580738; this version posted December 2, 2024. The copyright holder for this preprint (which was not certified by peer review) is the author/funder. This article is a US Government work. It is not subject to copyright under 17 USC 105 and is also made available for use under a CC0 license.

Supplementary Figures

Fig. S1. Bilateral MRI scans. Images from MRI scans of each marmoset representing coronal

bioRxiv preprint doi: https://doi.org/10.1101/2024.02.17.580738; this version posted December 2, 2024. The copyright holder for this preprint (which was not certified by peer review) is the author/funder. This article is a US Government work. It is not subject to copyright under 17 USC 105 and is also made available for use under a CC0 license.

planes ranging from pregeniculate region until retrosplenial area. Each slice is 0.25mm apart. ACC1 shows a unilateral lesion depicted by the arrow. CON2* and ACC3* are twins, and CON3# and ACC4# are twins.

Fig. S2. Representative images of white matter tract from a CON and ACC-lesioned subject. Both anterior and posterior corpus callosum are visibly narrowed in the area of the ACC lesion. The transverse widths of the white matter tracts were measured from sections stained for myelin at the following approximate rostrocaudal planes (in reference to the interaural axis): genu of the corpus callosum and rostrum of the corpus callosum (both +12.80 mm AP) were measured at the nadir of the overlaying cortex; anterior corpus callosum was measured at the midline and the anterior commissure was measured at the medial juncture with the internal capsule (both +9.50 mm AP); posterior corpus callosum and fimbriae were both measured at the junction with each other (both +1.80 mm AP).

Fig S3. Physical factors in developing marmosets**. (A)** Muscular strength was quantified using the Bioseb (BIO-GS4) grip strength monitor. The infant was allowed to grip onto a bar while being gently pulled backwards in a horizontal plan to determine the maximal peak force (grams). The ACC lesion did not cause any changes to forelimb muscular strength. **(B)** Average body weight (grams) with increasing age. Animals in both groups showed comparable body weights with increasing age. CON (n=5), ACC (n=5).

Figure S1

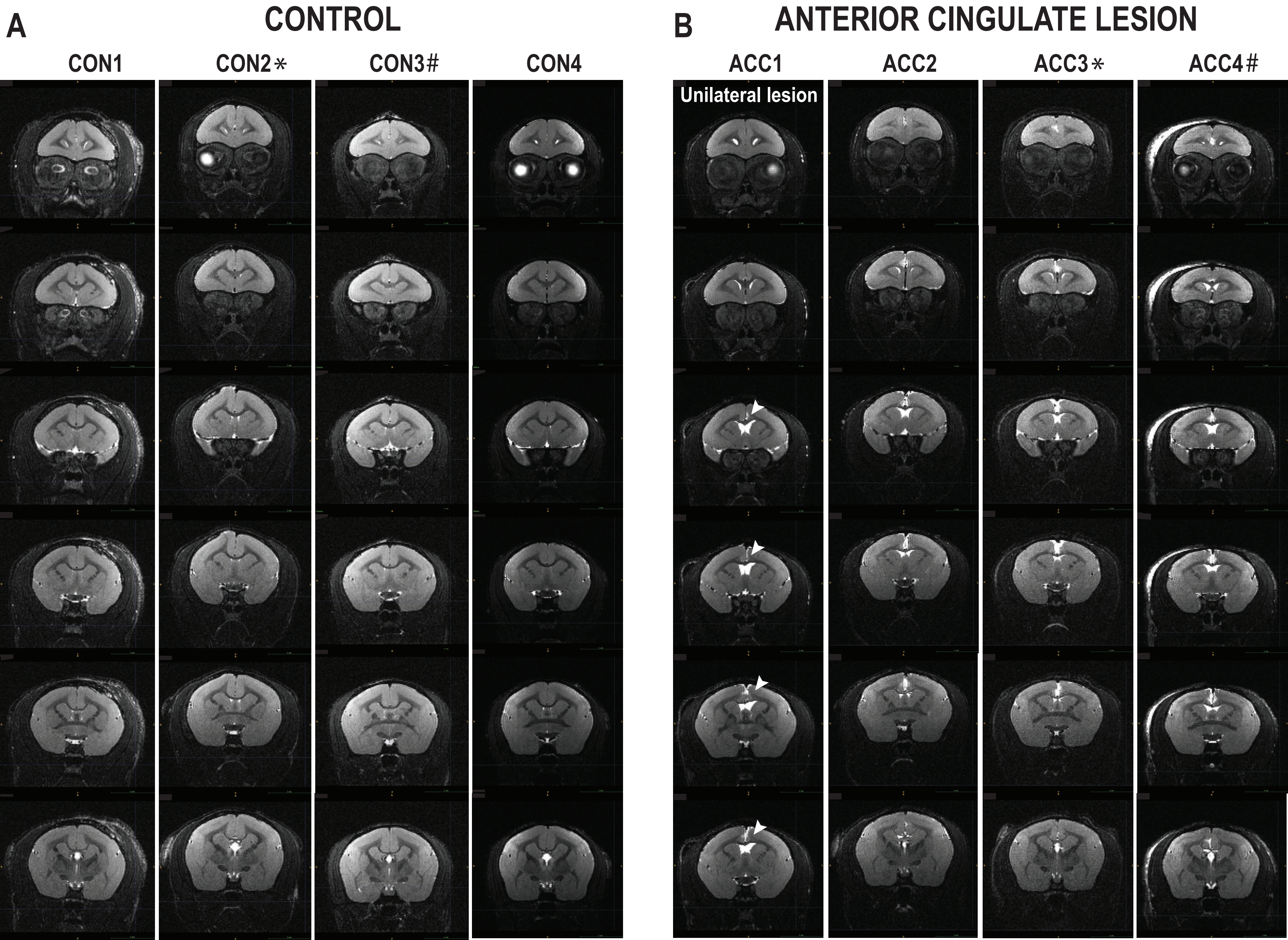
A
CONTROL
CON1
CON2*
CON3#
CON4
B
ANTERIOR CINGULATE LESION
ACC1
ACC2
ACC3*
ACC4#
Unilateral lesion

Figure S2

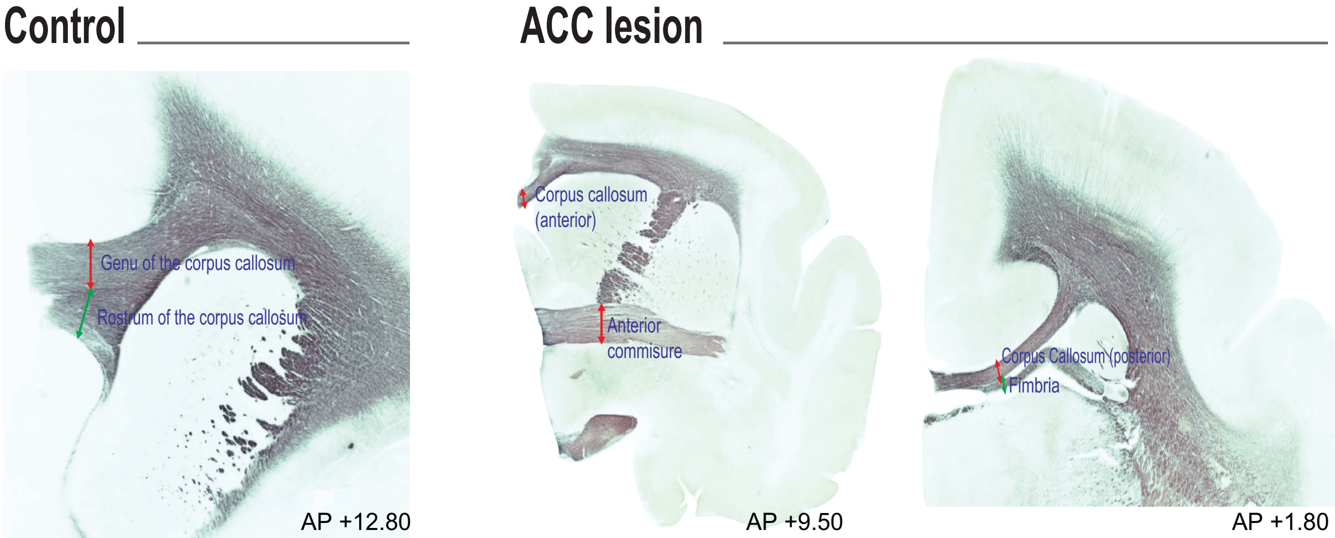
Control
ACC lesion
Genu of the corpus callosum
Rostrum of the corpus callosum
AP +12.80
Corpus callosum (anterior)
Anterior commisure
AP +9.50
Corpus Callosum (posterior)
Fimbria
AP +1.80

Figure S3

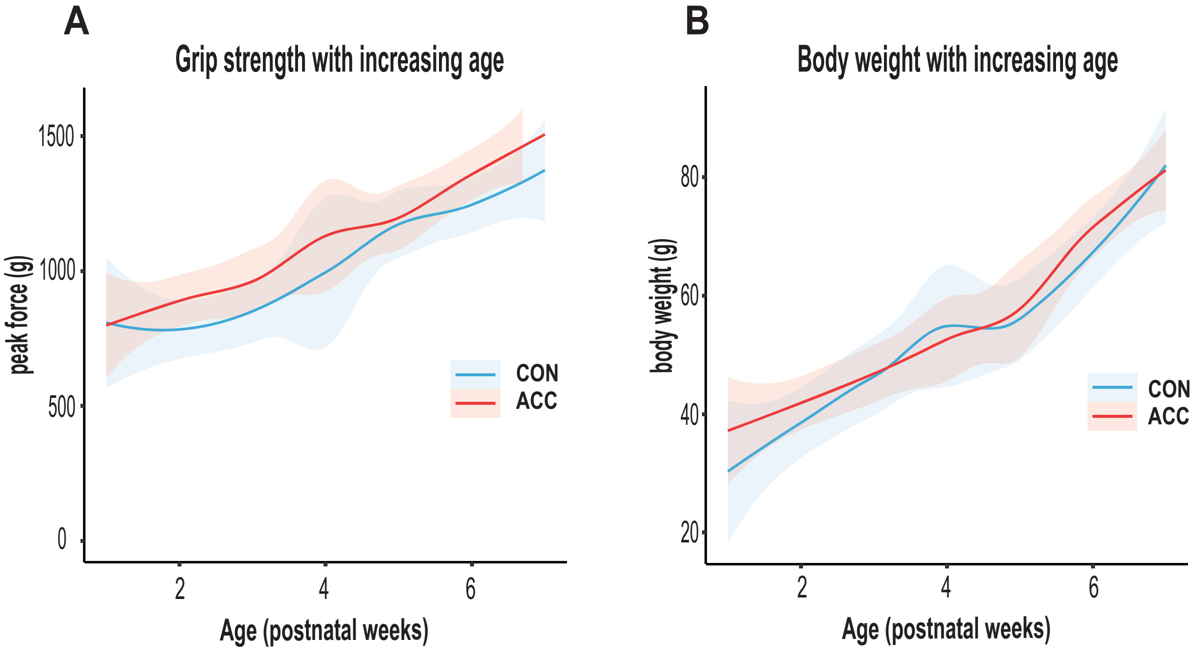
A
Grip strength with increasing age
peak force (g)
1500
1000
500
0
2
4
6
Age (postnatal weeks)
CON
ACC
B
Body weight with increasing age
body weight (g)
80
60
40
20
2
4
6
Age (postnatal weeks)
CON
ACC
